# Vascular access complications after catheter ablation of ventricular arrhythmias: Impact of vascular closure devices

**DOI:** 10.1016/j.hrthm.2024.09.001

**Published:** 2024-09-06

**Authors:** Chadi Tabaja, Nolan Hight, Arwa Younis, Shada Jadam, Joe Demian, Ayman Hussein, Jakub Sroubek, Walid Saliba, Mohamed Kanj, Mandeep Bhargava, Bryan Baranowski, Thomas Callahan, Mina Chung, Thomas Dresing, Justin Lee, Koji Higuchi, Ioan Liuba, David Martin, Shady Nakhla, John Rickard, Niraj Varma, Tyler Taigen, Oussama Wazni, Pasquale Santangeli

**Affiliations:** Cardiac Electrophysiology Section, Department of Cardiovascular Medicine, Cleveland Clinic, Cleveland, Ohio.

**Keywords:** Catheter ablation, Outcomes, Vascular access closure, Vascular complications, Ventricular arrhythmias

## Abstract

**BACKGROUND:**

Vascular access site complications are the most frequent complications of percutaneous catheter ablation (CA) of ventricular arrhythmias (VAs). Whether arterial/venous vascular closure devices (VCDs) prevent vascular complications is unknown.

**OBJECTIVE:**

We investigated the benefit of VCDs in patients undergoing CA of VAs.

**METHODS:**

Consecutive CA of VAs were included (2018–2022). Vascular accesses were obtained with ultrasound guidance. At the discretion of the operator, arterial and/or venous VCDs were used. Cases were divided into 3 groups: no use of VCDs for any arterial/venous accesses (manual compression [MC]), use of VCDs for some but not all accesses (Partial-VCDs), and use of VCDs for all accesses (Complete-VCDs). Vascular complications were classified as minor if they did not require intervention or major if they required intervention.

**RESULTS:**

A total of 1016 procedures were performed in 872 patients (mean age 62 ± 13 years; mean body mass index 30 ± 6 kg/m^2^; 27% female) during the study period. Femoral arterial access was obtained in 887 procedures (875 single access: 7.4 ± 1.5 F size; 12 two accesses: 7.3 ± 3 and 6.9 ± 1.8 F). Femoral venous access was obtained in 1014 procedures (unilateral in 17%; bilateral in 83%; mean number of access sites per patient 2.6 ± 0.7; mean size 8.4 ± 1.3 F). Hemostasis was achieved with MC in 192 procedures (19%), with Partial-VCD in 275 (27%), and with Complete-VCD in 549 (54%). A vascular complication occurred in 52 procedures (5.1%), including a minor hematoma in 3.9% and/or a major complication in 1.7%. The rate of vascular complications was 6.8% (5.2% minor and 1.6% major) in the MC group, 7.6% (5.1% minor and 3.3% major) in the Partial-VCD group, and 3.3% (2.9% minor and 0.9% major) in the Complete-VCD group (*P* = .014 for comparison). In multivariable analysis, Complete-VCD remained independently associated with a lower risk of vascular complications (odds ratio 0.69; 95% confidence interval 0.48–0.96; *P* = .036).

**CONCLUSION:**

In patients undergoing CA of VAs, Complete-VCD is associated with lower rates of vascular-related complications compared with MC or Partial-VCD.

## Introduction

In patients undergoing percutaneous catheter ablation (CA) of ventricular arrhythmias (VAs), the need for multiple arterial and venous vascular access points with large bore sheaths predisposes to vascular complications. Multiple studies have documented that vascular complications are the predominant adverse events of CA of VAs, with an incidence rate of major complications of up to 4% and a substantial impact on procedural morbidity, patient comfort, and health care costs.^1,2^ Although manual compression (MC) has been widely implemented as the standard of care for achieving hemostasis after femoral vascular access, the prolonged postprocedure bed rest and risk of rebleeding due to inadequate/insufficient compression are limitations of this approach. Vascular closure devices (VCDs) have emerged as an alternative strategy to establish quicker and more reliable hemostasis, although data supporting the beneficial role of VCDs for patients undergoing electrophysiological procedures are limited to femoral transvenous procedures such as CA of atrial fibrillation (AF), with studies suggesting noninferiority in terms of vascular access site complications, shorter time to hemostasis and bed rest, and improved patient comfort.^3^ Whether VCDs may be beneficial in patients undergoing CA of VAs, which often require a combination of arterial and venous vascular accesses, is unknown. In this study, we sought to evaluate the outcomes of arterial/venous VCDs relative to MC in patients undergoing CA of VAs.

## Methods

### Study population

We retrospectively analyzed data from consecutive patients undergoing CA of VAs (ventricular tachycardia [VT] or premature ventricular contraction [PVC]) at our institution between October 2018 and October 2022. Clinical data, procedural findings, and outcomes including all complications occurring within the first 30 days of the procedure were prospectively collected and adjudicated in a maintained registry. This study was approved by Cleveland Clinic’s Institutional Review Board.

### Vascular access management and procedural approach

Patients presented to the cardiac electrophysiology laboratory in the fasting state. For patients on oral anticoagulant therapy, oral anticoagulation was typically discontinued for 1–2 days before the procedure (for direct oral anticoagulants) or to achieve an international normalized ratio of <2 in patients on warfarin therapy. Heparin bridging in patients off oral anticoagulation was typically used in the presence of mechanical heart valves or in patients with permanent AF and estimated high thromboembolic risk (ie, CHA_2_DS_2_-VASc score ≥ 3). Chronic antiplatelet therapy was not discontinued. All vascular accesses were obtained with the Seldinger technique and with vascular ultrasound guidance (5- to 10-MHz linear array transducer with a maximum imaging depth of 6 cm) targeting the common femoral artery/vein in all cases. For patients undergoing common femoral arterial access, fluoroscopy was also used to mark the location of the center of the femoral head, which was used as a target for femoral arterial cannulation after validating the location of the bifurcation of the superficial femoral and profunda femoris arteries on ultrasound. A second access in the contralateral femoral artery was inserted whenever there was a high perceived risk of periprocedural hemodynamic decompensation with the potential (or planned) need for a hemodynamic support device. Arterial accesses were connected to a pressure transducer for arterial pressure monitoring.

Intravenous heparin with a target activated clotting time of >250 seconds was used during left ventricular endocardial mapping, whereas a single bolus of 5000 IU of heparin was typically used for procedures limited to the right ventricle (repeated in case of a prolonged procedure). At the end of the procedure, heparin was fully reversed with protamine (unless in the presence of mechanical heart valves, where heparin was only partially reversed) with a target activated clotting time of <170seconds. At the discretion of the operator, MC or VCDs were used to achieve arterial and/or venous access hemostasis. MC was performed by trained personnel. The duration of MC depended on the sheath size (in French) targeting a minimum of 3 minutes per each size (in French); for example, MC for an 8-F sheath required a minimum of 24 minutes (3 minutes × 8) of MC. In case of successful VCD, MC was held for 3 minutes (venous access) to 5 minutes (arterial access). Bleeding at the access site was reassessed periodically, and supplemental MC was performed in case of persistent bleeding.

VCDs included Perclose Proglide (Abbott Vascular, Chicago, IL) or Angio-Seal (Terumo Medical, Tokyo, Japan) for arterial access closure and/or a figure-of-eight nonresorbable suture (removed 4 hours postprocedure) or a Vascade MVP (Haemonetics, Clinton, PA) device for venous access closure. A Perclose Proglide (Abbott Vascular) device was used for 5 venous access closures (range access size 8–11 F).

The recommended duration of bed rest was 6 hours whenever arterial access was obtained and regardless of the use of VCD. For procedures using only femoral venous accesses, the recommended bed rest was 3 hours when complete access closure was obtained, otherwise 4–5 hours. A pressure bandage was applied at the discretion of the operator.

### End point definition

The end point of interest was a composite of vascular access site major and minor vascular complications within 30 days of the index procedure. Vascular complications were classified as minor if they did not require any intervention or major if they led to significant bleeding (≥3 g/dL drop in hemoglobin) or required additional intervention such as blood transfusion, percutaneous, or surgical repair. These complications included vascular pseudoaneurysm, hematoma at the access site with significant bleeding, deep vein thrombosis, arterial thrombosis, new-onset limb ischemia, permanent access site–related nerve injury, and arteriovenous fistula.

Patients were divided into 3 groups according to the use of VCDs:

1. MC: No use of VCDs for any arterial/venous accesses with hemostasis achieved with MC only. This group also included patients in whom VCDs were attempted but acutely failed and converted to MC for all accesses.
2. Partial-VCD: Successful use of VCDs for some but not all arterial/venous accesses, with MC planned and used for remaining accesses.
3. Complete-VCD: Successful use of VCDs for all arterial/venous accesses.

### Statistical analysis

Continuous data are reported as mean ± SD or median (25th–75th percentile) for skewed distributions. Categorical data are reported as number (percentage). The unpaired Student *t* test, Mann-Whitney *U* test, or Kruskal-Wallis *H* test, when appropriate, were used to determine differences between groups for continuous variables. The *χ*^2^ test was used to compare differences across groups for categorical variables. Univariate and multivariable logistic regression analyses were applied for the assessment of the association of baseline clinical and procedural variables and approach used for vascular hemostasis with the occurrence of vascular complications. Relative risk estimates for vascular complications were reported as odds ratios (ORs) and 95% confidence intervals (CIs). A *P* level of <.05 was considered to indicate statistical significance. Statistical analyses were performed by using Stata version 18 (Stata Corporation, College Station, TX).

## Results

### Clinical characteristics and periprocedural data

During the study period, a total of 1016 CA procedures were performed in 872 patients (mean age 62 ± 13 years; mean body mass index 30 ± 6 kg/m^2^; 27% female). Of these, 592 (58%) were CA of PVCs and 424 (42%) CA of VT. A total of 303 procedures (30%) were performed in patients who were on chronic oral anticoagulant therapy (37% on warfarin and 63% on direct oral anticoagulants), which were discontinued before the procedure in 297 of 303 cases (98%). The 6 patients in whom oral anticoagulation was not discontinued (3 on apixaban and 3 on rivaroxaban) had AF and CHA_2_DS_2_-VASc score ≥ 5 and underwent transfemoral venous access–only procedures. A total of 522 procedures (51%) were performed in patients on chronic antiplatelet therapy (aspirin in 48%, clopidogrel in 11%, and others including ticagrelor or prasugrel in 2.2%). Antiplatelet agents were not discontinued before the procedure.

Arterial access was obtained in 887 procedures (87%). Of these, 875 were single access of the right common femoral artery, with a mean access size of 7.4 ± 1.5 F (range 4–15 F), and 12 were bilateral common femoral artery accesses (mean size 7.3 ± 3 F for the right femoral artery access and 6.9 ± 1.8 F for the left femoral artery access). Femoral venous access was obtained in 1014 procedures (99%). A total of 2605 separate venous accesses (unilateral in 17% and bilateral in 83%) were obtained with a mean of 2.6 ± 0.7 access sites per patient and a mean size of 8.3 ± 1.3 F.

Overall, 129 procedures (13%) were performed via a transfemoral venous access–only route and 2 (0.2%) with a transfemoral arterial access–only approach. Vascular hemostasis was achieved with MC in 192 procedures (19%), with Partial-VCD in 275 (27%), and with Complete-VCD in 549 (54%). In the Partial-VCD group, a VCD was used for the arterial access and MC for the venous access in two-thirds of the cases. In the remaining third, operators opted to use MC for arterial accesses (most commonly for sheath access sizes <8 F) and a VCD for the venous accesses.

**Figure 1:**
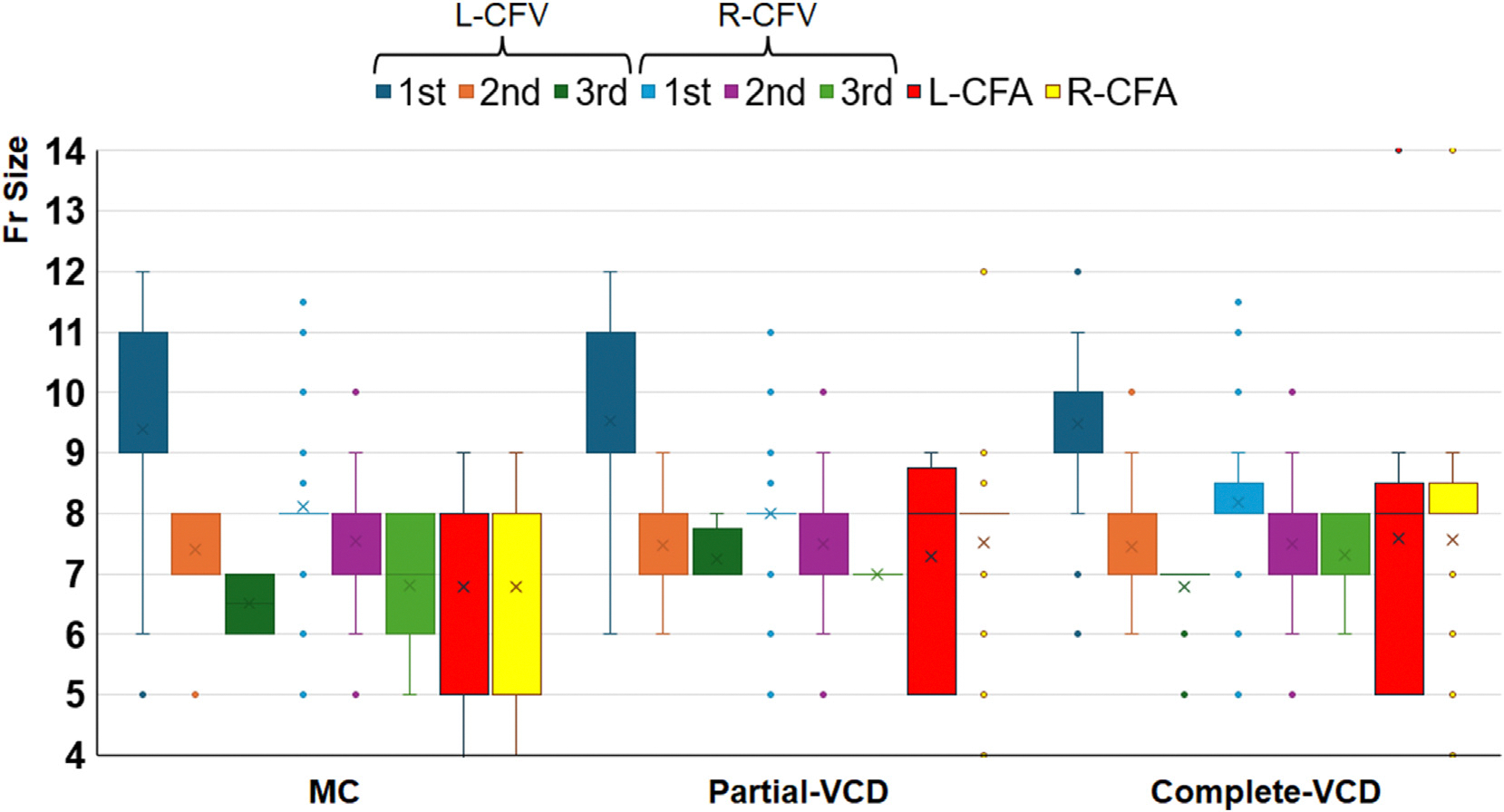
Distribution of sheath sizes (in French) across 3 study groups. L-CFA = left common femoral artery; L-CFV = left common femoral vein; MC = manual compression; R-CFA = right common femoral artery; R-CFV = right common femoral vein; VCD = vascular closure device.

Complete-VCD cases were more often on chronic oral anticoagulation therapy before the procedure (more commonly with direct oral anticoagulants), more frequently received femoral arterial access (compared to the MC group), and had a higher number of femoral venous accesses with larger venous sheaths (Tables 1 and 2). The overall distribution of sheath sizes for venous and arterial vascular accesses is presented in Figure 1. Of note, the Complete-VCD group had significantly larger average arterial and venous sheath sizes than did the other groups. A pressure bandage was used in 304 cases (30%), more commonly in the MC group (34%) and Partial-VCD group (37%) vs Complete-VCD group (25%) (*P* < .001 for comparison).

An indwelling bladder catheter was used in a total of 506 procedures (50%), most commonly for VT ablation procedures (63% vs 41% of PVC ablation procedures; *P* < .001) and similarly distributed across the 3 study groups (44% in the MC group, 52% in the Partial-VCD group, and 51% in the Complete-VCD group; *P* = .22 for comparison). A urinary complication (ie, urinary tract infection or persistent urinary retention) occurred in 13 cases (1.3%) and was more frequent when an indwelling bladder catheter was used (11 [2.2%] in patients who received an indwelling bladder catheter vs 2 [0.4%] in patients who did not receive an indwelling bladder catheter; *P* = .011). The duration of hospital stay was similar between patients who experienced a urinary complication and those who did not (median 3.5 days; interquartile range 2.5–4 days vs 3.5 days; interquartile range 2.5–4.5 days; *P* = .57).

### Vascular complications

**Figure 2:**
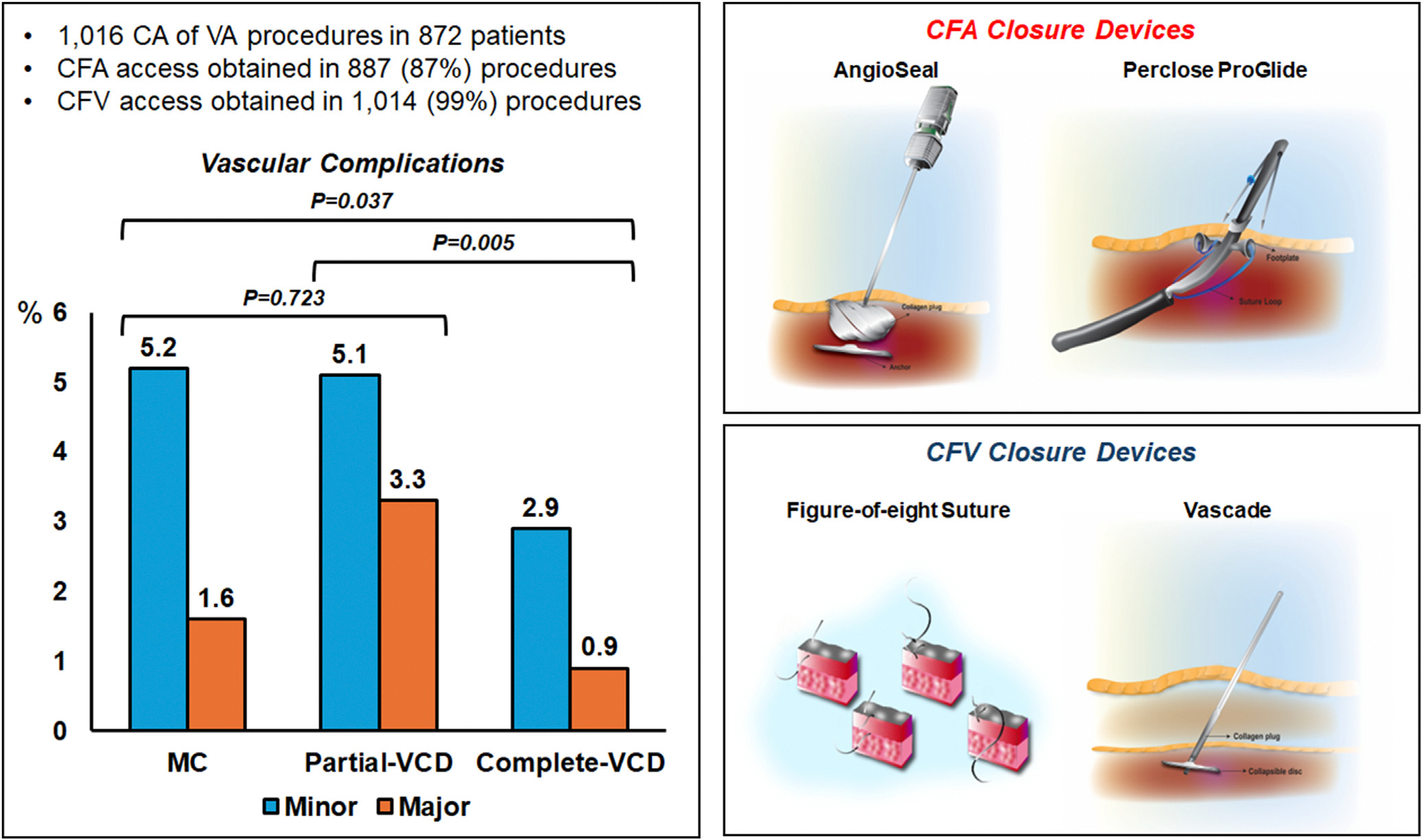
Summary of the main study findings. CA = catheter ablation; CFA = common femoral artery; CFV = common femoral vein; MC = manual compression; VA = ventricular arrhythmia; VCD = vascular closure device.

A vascular access–related complication occurred in 52 procedures (5.1%). This included a minor complication in 3.9% (ie, minor hematomas not requiring intervention) and/or a major complication in 1.7%. The type of major vascular complications is reported in Table 3. Overall, the rate of vascular complications was 6.8% (5.2% minor and 1.6% major) in the MC group, 7.6% (5.1% minor and 3.3% major) in the Partial-VCD group, and 3.3% (2.9% minor and 0.9% major) in the Complete-VCD group (*P* = .014 for comparison) (Figure 2). Table 4 compares the clinical and procedural characteristics in patients with and without vascular complications. The total number of femoral arterial accesses was the only significant univariate predictor of increased risk of vascular complications (OR 3.57; 95% CI 1.29–9.86; *P* = .014). Univariate predictors of lower risk of vascular complications included total number of femoral venous accesses (OR 0.62; 95% CI 0.41–0.92; *P* = .017), use of a femoral venous access–only approach (OR 0.05; 95% CI 0.01–0.86; *P* = .039), and Complete-VCD (OR 0.66; 95% CI 0.47–0.92; *P* = .017).

In multivariable analysis, after adjusting for age, sex, body mass index, use of antiplatelet agents or oral anticoagulants, number of arterial accesses, and number of femoral venous accesses, Complete-VCD remained independently associated with a lower risk of vascular complications (OR 0.69; 95% CI 0.48–0.96; *P* = .036). The specific type of VCD used (Perclose Proglide vs Angio-Seal for arterial closure and figure-of-eight nonresorbable suture vs Vascade MVP for venous closure) did not appear to influence the risk of vascular complications.

Other independent predictors of vascular complications were the number of arterial accesses that increased risk (OR 3.19; 95% CI 1.14–8.94; *P* = .027) and the number of venous accesses that decreased risk (OR 0.64; 95% CI 0.43–0.96; *P* = .032). In line with these findings, vascular complications occurred in only 1 of 129 (0.78%; minor groin hematoma not requiring intervention) procedures performed via a transfemoral venous route only (ie, no arterial access) vs 51 of 887 (5.8%; including all major vascular complications occurring in the study) procedures with at least 1 arterial access performed. Patients with vascular complications had a significantly longer duration of hospital stay (median 3 days; interquartile range 2–9 days vs 2; interquartile range 2–7 days; *P* = .002).

Finally, 30-day mortality occurred in 29 cases (2.9%) (5.4% after VT ablation and 1% after PVC ablation). Vascular complications did not appear to influence 30-day mortality (OR 1.4; 95% CI 0.32–6.01; *P* = .661).

## Discussion

This study was designed to appraise the impact of different strategies to achieve postprocedural vascular hemostasis in a large contemporary cohort of patients undergoing CA of VAs, and it mainly documents a significantly lower rate of vascular access–related complications associated with successful complete arterial and venous access closure with VCDs.

Vascular access complications remain the most common complications of CA of VAs with a substantial impact on morbidity, need for additional unplanned interventions, and health care costs.^2,4^ In these patients, multiple complex comorbidities are typically coexistent, and the frequent need for multiple access points (venous and/or arterial) for mapping and ablation can predispose to a higher risk of vascular complications. To our knowledge, no prior study has systematically evaluated predictors of vascular complications and best approaches to minimize them in patients undergoing CA of VAs. Our study supports the benefit of VCDs relative to MC in these patients if complete vascular closure (venous and arterial) is successfully accomplished, with an overall similar magnitude of risk reduction for both minor (43% relative risk reduction) and major (64% relative risk reduction) complications. Importantly, these results occurred in the context of more use of chronic oral anticoagulation therapy before the procedure in the Complete-VCD group (compared with the MC group), with a high usage of femoral arterial access, higher number of femoral venous accesses, and overall larger sheath sizes. On the contrary, the benefit of VCDs was evident only in the case of complete access closure (venous and arterial), as the rate of vascular complications was not significantly different between the MC group and the Partial-VCD group in our study (Figure 1).

These findings are in line with prior studies on different interventional/structural cardiology procedures that, similarly to VA ablation, require ≥1 arterial access with large bore sheaths and have shown that complete access closure is associated with a reduced rate of vascular complications.^5–7^ In this context, we found that the total number of arterial accesses was also an independent predictor of vascular complications whereas femoral venous access (and number of accesses) appeared protective. The latter finding was likely multifactorial. First, we observed excellent outcomes in the 129 procedures performed via a transfemoral venous access–only route (ie, no arterial access). In this subgroup, an overall higher number of venous access points was obtained compared with patients in whom arterial access was obtained (2.8 ± 0.8 venous accesses vs 2.5 ± 0.7 venous accesses; *P* = .001 for comparison) and no major vascular complications occurred, with only 1 case of minor groin hematoma not requiring intervention. Of note, these findings have modified our current institutional approaches to patients undergoing CA of VAs, in whom femoral arterial access is avoided whenever possible, and a transvenous femoral route only is used as a first-line strategy in order to minimize the risk of vascular complications. Furthermore, within the group of patients who had any arterial access obtained, those with arterial accesses <8 F had a higher number of femoral venous accesses than did those who had arterial accesses ≥8 F (2.8 ± 0.7 venous accesses vs 2.4 ± 0.7 venous accesses; *P* < .001 for comparison). These figures may explain the protective effect of the increasing number of venous accesses seen in our analysis.

Notably, all vascular accesses were performed under ultrasound guidance, which has also been shown to reduce the rate of vascular complications, particularly those related to inadvertent vascular side branch puncture and/or arteriovenous fistula.^8–10^ In this regard, we did not observe any case of arteriovenous fistula, a finding likely explained by the systematic use of vascular ultrasound guidance. However, while ultrasound guidance is crucial for a proper understanding of the vascular anatomy and the relationship between the common femoral artery and the vein, it may not be enough to prevent vascular bleeding complications for procedures that require large bore arterial access.^11^ In these cases, a proper hemostasis technique may be more relevant to prevent complications, and our study suggests that VCDs may provide benefit in this sense in adjunct to vascular ultrasound guidance.

Finally, procedural cost is an important consideration when systematic VCD use for all arterial and venous accesses is considered. While our study did not evaluate the cost-effectiveness of VCDs in patients undergoing CA of VAs, the need for additional in-hospital interventions and the significantly prolonged hospital stay in patients experiencing vascular complications may offset the initial increased cost of VCDs. On the contrary, 30-day postprocedural mortality did not seem affected by the occurrence of vascular complications, which is in line with prior investigations.^12^ Additional studies are needed to address whether the systematic use of VCDs to achieve complete arterial and venous closure in patients undergoing CA of VAs is a cost-effective strategy. In this regard, as the specific type of VCD used did not appear to influence the risk of vascular complications, our current clinical practice for femoral venous access closure has been modified to prefer the more cost-effective figure-of-eight suture over the Vascade MVP device.

### Limitations

This study has limitations inherent to its observational retro-spective design. The use of VCDs was discretional, and the specific reasons that prompted a given operator to use a VCD or MC for vascular access were not retrievable. The type and size of the needle used for vascular accesses were not collected. In this regard, there is evidence that the use of a 21-G micro-puncture needle may reduce vascular complications compared to a standard 18-G needle in patients undergoing femoral arterial access.^13^ Information on postprocedural pain at the access site was also not systematically collected. The MC group included patients in whom a VCD was attempted but failed and were ultimately managed with MC. This specific subset may be at increased risk of vascular complications compared with patients in whom MC was used as a planned initial strategy (ie, not bailout).^14^ Similarly, the Partial-VCD group consisted of patients in whom VCDs were intentionally used for some but not all arterial/venous accesses, with MC used for remaining accesses. As often >1 access per side was performed, it is difficult to determine whether a given complication occurring in this group (eg, groin hematoma) was related to the access that received a VCD vs the one that received MC. Finally, only 2 types of arterial closure devices (Perclose Proglide and Angio-Seal) and venous closure devices (figure-of-eight nonresorbable suture and Vascade MVP) were used in our study and, as mentioned, there appeared to be no differences in outcomes when comparing different VCDs. Whether these results can be generalized to other types of VCDs warrant further investigation.

## Conclusion

In patients undergoing CA of VAs, complete arterial and venous access closure with VCDs is associated with a lower risk of vascular-related complications compared to MC or Partial-VCD.

## Figures and Tables

**Table 1:** Comparison of baseline clinical characteristics across different study groups

Characteristic	Vascular hemostasis group			*P*
	MC (n = 192)	Partial-VCD (n = 275)	Complete-VCD (n = 549)	

Age (y)	61 ± 15	61 ± 13	63 ± 12	.06
BMI (kg/m^2^)	29 ± 6	30 ± 6	30 ± 6	.94
Sex: female	141 (73)	199 (72)	408 (74)	.83
CAD	68 (35)	99 (36)	221 (40)	.34
Diabetes	42 (22)	59 (22)	92 (17)	.14
Dialysis	3 (1.6)	6 (2.2)	16 (2.9)	.55
Hypertension	103 (54)	153 (56)	286 (52)	.58
PAD	8 (4)	7 (3)	15 (3)	.54
LVEF (%)	41 ± 15	42 ± 14	41 ± 15	.69
Antiplatelet therapy	88 (46)	148 (54)	286 (52)	.21
Single Antiplatelet	72 (38)	122 (45)	238 (43)	.54
Dual antiplatelet	16 (8)	26 (9)	48 (9)	.54
Type of antiplatelet				
Aspirin	80 (42)	139 (50)	268 (49)	.15
Clopidogrel	21 (11)	28 (10)	61 (11)	.92
Others	3 (1.6)	6 (2.2)	5 (0.9)	.48
OAC therapy	48 (25)	73 (27)	182 (33)	.040
Type of OAC				.030
Warfarin	26/48 (54)	24/73 (33)	61/182 (33)	-
Apixaban	15/48 (31)	38/73 (52)	92/182 (51)	-
Rivaroxaban	6/48 (13)	10/73 (14)	29/182 (16)	-
Dabigatran	1/48 (1)	1/73 (1)	0/182 (0)	-

Values are presented as mean ± SD or n (%).

BMI = body mass index; CAD = coronary artery disease; LVEF = left ventricular ejection fraction;MC = manual compression; PAD = peripheral artery disease; OAC = oral anticoagulant; VCD = vascular closure device.

**Table 2:** Comparison of main procedural characteristics across different study groups

Characteristic	Vascular hemostasis group			*P*
	MC (n = 192)	Partial-VCD (n = 275)	Complete-VCD (n = 549)	

VT ablation	76 (40)	115 (42)	233 (42)	.78
PVC ablation	116 (60)	160 (58)	316 (58)	.78
Arterial access	151 (79)	275 (100)	461 (84)	<.001
Right CFA	140 (73)	269 (98)	453 (82)	<.001
Left CFA	17 (9)	9 (3)	11 (2)	<.001
Both	6 (3)	3 (1)	3 (0.5)	.017
Venous access	192 (100)	275 (100)	547 (99)	.42
No. of venous accesses*	2 (2–3)	2 (2–3)	3 (2–3)	.048
No. of right CFVs*	1 (1–2)	1 (1 –2)	1 (1–2)	.013
Access size right CFV (F)	7.9 ± 0.9	7.9 ± 0.9	8.1 ± 0.9	.026
No. of left CFVs*	1 (1–1)	1 (1–2)	1 (1–2)	<.001
Access size left CFV (F)	8.9 ± 1.6	8.9 ± 1.5	8.8 ± 1.4	.15
Procedure duration (h)	3.6 ± 1.4	3.9 ± 1.5	3.4 ± 1.3	.010

Values are presented as mean ± SD or n (%).

CFA = common femoral artery; CFV = commonfemoral vein; MC = manual compression; PVC = prematureventricularcontraction;VCD = vascularclosure device; VT = ventricular tachycardia.

* Data are presented as median (interquartile range).

**Table 3:** Type of vascular complications across different study groups

Complications	Vascular hemostasis group			*P*
	MC (n = 192)	Partial-VCD (n = 275)	Complete-VCD (n = 549)	

Minor complication	10 (5.2)	14 (5.1)	16 (2.9)	.19
Minor hematoma	10 (5.2)	14 (5.1)	16 (2.9)	.19
Major complication	3 (1.6)	9 (3.3)	5 (0.9)	.04
Bleeding requiring transfusion	1 (0.5)	4 (1.5)	3 (0.5)	.34
Arterial pseudoaneurysm	1 (0.5)	5 (1.8)	1 (0.2)	.03
Deep vein thrombosis	0 (0)	0 (0)	1 (0.2)	.65
Ischemic limb	1 (0.5)	0 (0)	0 (0)	.12

Values are presented as n (%).

MC = manual compression; VCD = vascular closure device.

**Table 4:** Comparison of clinical and main procedural characteristics in patients with and without vascular complications

Characteristic	No vascular complications (n = 964)	Vascular complications (n = 52)	*P*

Clinical characteristics			
Age (y)	62 ± 13	64 ± 12	.29
BMI (kg/m^2^)	30 ± 6	30 ± 5	.66
Sex: female	255 (26)	13 (25)	.82
CAD	367 (38)	21 (40)	.76
Diabetes	184 (19)	9 (17)	.74
Dialysis	23 (2.4)	2 (3.9)	.51
Hypertension	512 (53)	30 (58)	.53
PAD	28 (2.9)	2 (3.9)	.69
LVEF (%)	41 ± 15	44 ± 14	.22
Antiplatelet therapy	498 (52)	24 (46)	.44
Single antiplatelet	415 (43)	17 (33)	.36
Dual antiplatelet	83 (9)	6 (12)	.36
OAC therapy	286 (30)	17 (33)	.64
Procedural characteristics			
Arterial access	836 (87)	51 (98)	.017
Right CFA	812 (84)	50 (96)	.020
Left CFA	35 (4)	2 (4)	.94
Both	11 (1)	1 (2)	.61
Total no. of CFA accesses	0.9 ± 0.4	1 ± 0.2	.015
Venous access	963 (99.9)	51 (98)	.004
No. of venous accesses*	3 (2–3)	2 (2–3)	.045
No. of right CFVs*	1 (1–2)	1 (1 –2)	.68
Access size right CFV (F)	7.9 ± 0.9	7.9 ± 0.9	.48
No. of left CFVs*	1 (1–2)	1 (1–1)	.026
Access size left CFV (F)	8.9 ± 1.4	8.9 ± 1.6	.92
Procedure duration (h)	3.6 ± 1.4	3.9 ± 1.6	.09
Hospital stay (d)*	2 (2–7)	3 (2–9)	.002

Values are presented as mean ± SD or n (%).

BMI = body mass index; CAD = coronaryarterydisease; CFA = common femoral artery; CFV = commonfemoral vein; LVEF = leftventricularejection fraction; PAD = peripheral artery disease; OAC = oral anticoagulant.

* Data are presented as median (interquartile range).

## Abbreviations

CA: catheter ablation
MC: manual compression
VA: ventricular arrhythmias
VCD: vascular closure devices
